# Analysis of SHIP1 expression and activity in Crohn’s disease patients

**DOI:** 10.1371/journal.pone.0182308

**Published:** 2017-08-02

**Authors:** Rajesh Somasundaram, Sandra Fernandes, Jasper J. Deuring, Colin de Haar, Ernst J. Kuipers, Lauran Vogelaar, Frank A. Middleton, C. Janneke van der Woude, Maikel P. Peppelenbosch, William G. Kerr, Gwenny M. Fuhler

**Affiliations:** 1 Department of Gastroenterology and Hepatology, Erasmus MC, University Medical Center Rotterdam, ‘s Gravendijkwal 230, Rotterdam, the Netherlands; 2 Department of Microbiology & Immunology, SUNY Upstate Medical University, Syracuse, NY, United States of America; 3 Department of Neuroscience and Physiology, SUNY Upstate Medical University, Syracuse, NY, United States of America; 4 Department of Pediatrics, SUNY Upstate Medical University, Syracuse, NY, United States of America; Monash University, AUSTRALIA

## Abstract

**Background:**

SH2 domain containing inositol-5-phosphatase (SHIP1) is an important modulator of innate and adaptive immunity. In mice, loss of SHIP1 provokes severe ileitis resembling Crohn’s disease (CD), as a result of deregulated immune responses, altered cytokine production and intestinal fibrosis. Recently, SHIP1 activity was shown to be correlated to the presence of a CD-associated single nucleotide polymorphism in *ATG16L1*. Here, we studied SHIP1 activity and expression in an adult cohort of CD patients.

**Methods:**

SHIP1 activity, protein and mRNA expression in peripheral blood mononuclear cells from CD patients in clinical remission were determined by Malachite green assay, Western blotting and qRT-PCR respectively. Genomic DNA was genotyped for *ATG16L1* rs2241880.

**Results:**

SHIP1 protein levels are profoundly diminished in a subset of patients; however, SHIP1 activity and expression are not correlated to *ATG16L1* SNP status in this adult cohort.

**Conclusions:**

Aberrant SHIP1 activity can contribute to disease in a subset of adult CD patients, and warrants further investigation.

## Introduction

The SH2 domain-containing inositol-5’-phosphatase 1 (SHIP1), predominantly expressed in hematopoietic tissues [1], is required for the regulation of immune cell compartments, lymphoid and innate immune cell function and intestinal fibrosis [2,3].We and others have shown that SHIP1^-/-^ mice spontaneously develop an intestinal inflammatory phenotype resembling the inflammatory bowel syndrome (IBD) Crohn’s disease (CD), with increased granulocyte infiltration and a profound T cell depletion observed in the small intestine [4,5]. Development of a CD-like phenotype in SHIP1^-/-^ mice is dependent on hematopoietic cells, as transplantation of SHIP1^-/-^ hematolymphoid cells into wild type mice is sufficient to transfer ileitis and WT bone marrow transplantation cures SHIP1^-/-^ mice of mucosal inflammatory disease in both the lungs and small intestine [4,6]. Furthermore, while elective SHIP1 deficiency in the myeloid compartment alone is not sufficient to cause CD or pneumonia in SHIP1^-/-^ mice [7], dual lineage deletion of SHIP1 in both the T- and myeloid cell compartments causes CD-like disease [4]. The role of SHIP1 in human disease is as yet unclear. While enhanced SHIP1 mRNA expression was observed in biopsies from CD patients [8], Ngoh and colleagues recently demonstrated decreased SHIP1 protein expression and activity in peripheral blood mononuclear cells (PBMCs) and biopsies in a cohort consisting mainly of treatment-naive subjects with ileal CD [9,10]. Interestingly, this downregulation was correlated with the presence of a single nucleotide polymorphism (SNP) in the *ATG16L1* gene, one of the best described CD risk genes, which lies adjacent to the SHIP1 gene, *INPP5D*. Furthermore, SHIP1 activity was inversely correlated to the expression of IL1b, which contributes to disease severity. Genetic contribution to disease as well as disease behavior differs between cohorts [11]. In addition, treatments may affect SHIP1 activity and expression. We therefore analysed SHIP1 activity and expression in a daily practice cross-sectional cohort of adult patients in the Netherlands, to determine to what extent aberrant SHIP1 activity may contribute to CD pathology in these patients. Our data indicate that SHIP1 expression is abrogated in a small subset of CD patients and SHIP1 activity in general is decreased, but SHIP1 activity and expression are not correlated to *ATG16L1* status in this adult cohort of patients.

## Material and methods

### Patients

Heparin-anticoagulated blood was obtained from each subject using standard venipuncture technique, at the Erasmus University Medical Center, according to institutional guidelines (METC-2004-168). In total, 34 patients were included for SHIP1 activity assays (**Table 1**). Diagnosis of CD was established based on standard criteria, by clinical assessment, radiology, endoscopy and histology. Patients who were in clinical remission according to Harvey Bradshaw criteria, had low CRP levels, or did not receive intensification of treatment at the time of blood collection were eligible for inclusion. There were no other exclusion criteria. See **S1 Table** for individual patient characteristics.

**Table 1:** Patient characteristics (patients included in the SHIP activity assay).

Number, n	34
Mean age, yr (range)	37 (18–61)
Sex, n (%)	
- female	18 (53%)
- male	16 (47%)
Mean age at diagnosis, yr (range)	25 (10–55)
Mean duration of disease, yr (range)	12 (1–32)
Location, n (%)	
- terminal ileum (L1)	9 (26.5%)
- colon (L2)	10 (29.4%)
- ileocolon (L3)	15 (44.1%)
Disease behaviour	
- B1	15 (44.1%)
- B2	11 (32.4%)
- B3	8 (23.5%)
Resection, n (%)	16 (47%)
CRP (mg/L, mean±SEM)	3.4±1.09
Medication, n (%)	
- None	9 (26.5%)
- 5-ASA	5 (14.7%)
- Steroids	3 (8.8%)
- immunosuppressives	11 (32.3%)
- Anti-TNF	16 (47.1%)

### Phosphatase assay

For every experiment with CD patients, healthy donors were included in parallel as normal controls. Phosphatase assays were performed as earlier described [12]. PBMCs were isolated from Heparin-anticoagulated blood by Ficoll (Amersham, Upsala, Sweden) density centrifugation and lysed in IP-lysis buffer (20 mM Tris, 150 mM NaCl, 1 mM EDTA, 1 mM EGTA, 1% Triton X100, 1 mM PMSF, Halt protease inhibitor). Alternatively, OPM2, U266, MDA-MB-231 or MCF7 cells were washed with PBS, and lysed in IP-lysis buffer. Protein content was determined by RC/DC protein assay as per manufacturer’s directions (Bio-Rad Laboratories, Hercules CA). SHIP1 or OCRL-1 were immunoprecipitated from 200 μg protein using SHIP1 (P1C1) or OCRL (4E3) antibodies from Santa Cruz Biotechnology (Santa Cruz, CA) and Gammabind sepharose (GE healthcare, Diegem, Belgium). Beads were washed twice with IP lysis buffer and once with TBS/MgCl_2_ (10mM) and resuspended in TBS/MgCl_2._ Immunoprecipitated SHIP1 was incubated in the presence of 100μM PtdIns(3,4,5)P_3_ (Echelon Biosciences, Salt Lake City,UT) for 35 minutes, whereas immunoprecipitated OCRL1 was incubated with PtdIns(4,5)P_2_. Malachite Green solution was added according to manufacturer’s instructions, and the plate was read after 20 minutes. Background (OD640 of gammabind sepharose beads control) was subtracted from obtained OD640 values of immunoprecipitates to present the total cellular SHIP1 activity (per 200 μg protein). Human recombinant SHIP2 (0.025 μg/μl) was used as positive control for the Malachite Green reaction. As the amount of activity observed is dependent on the total input of SHIP1 protein into the immunoprecipitation, the intrinsic SHIP1 activity was determined by correcting the SHIP1 activity levels for SHIP1 expression levels in the lysate, as determined by quantitative Western blot analysis.

### Quantitative Western blot analysis

Immunoblotting of PBMC lysates was performed as earlier described, with some adjustments [13]. After preparation of lysates for immunoprecipitation, an aliquot of this same lysate was taken for Western blot analysis. Gels were blotted on Immobilon-FL transfer membrane (Millipore, Billerica, MA). Anti-rabbit or anti-mouse IRDye-conjugated secondary antibodies were used according to manufacturer’s directions, and blots were scanned by Odyssey infrared imaging (LI-COR Biosciences, Lincoln, NE). SHIP1 P1C1 and β-actin antibodies were from Santa Cruz Biotechnology, Santa Cruz, CA. SHIP2 and PTEN antibodies were from Cell Signaling Technology (Beverly, MA). Quantification was performed using Odyssey 3.0 software. To calculate the relative SHIP1, phospho-SHIP1, SHIP2 or PTEN levels, densitometric values of these phosphatases were divided by densitometric values of the β-actin signal in the same lanes.

### Cells and cell lines

PBMCs from concentrated lymphocyte fractions (Sanquin Bloodbank, Amsterdam, the Netherlands) were isolated by Ficoll density centrifugation and resuspended in PBS/0.5% bovine serum albumin (BSA)/2mM EDTA. Enrichment of T cells, B cells and monocytes was performed by magnetic activated cell sorting (MACS) through CD3 positive selection, CD19 positive selection and CD14 positive selection, respectively (Miltenyi Biotec, Auburn, CA), according to manufacturer’s protocol.

Multiple Myeloma (MM) cell lines RPMI8226, U266 and OPM2 (ATCC, Rockville, MD) were routinely maintained in Iscove’s Modified Dulbecco’s Medium (IMDM, ATCC, Rockville, MD) supplemented with 10% fetal calf serum (FCS, Mediatech, Manassas, VA), whereas MCF-7 and MDA-MB-231 breast cancer cells were cultured in Eagle’s Minimal Essential Medium (EMEM) with 10% FCS, L-glutamine and 0.1 mg/ml insulin.

### Immunohistochemistry

Cytocentrifuge preparations of isolated PBMCs or Caco2 cells (6x10^5^) were fixed in ice-cold acetone, and incubated overnight with SHIP1 antibody in PBS containing 5% goat serum and 0.05% Tween20 at 4°C. Slides were washed with PBS/0/05%Tween20 and incubated with horseradish peroxidase conjugated antibodies (Dako, Heverlee, Belgium). Peroxidase activity was demonstrated using 0.05M Tris-HCl buffer containing 0.5 mg/ml 3,3’-diaminobenzidine tetrahydrochloride (DAB, Sigma, St Louis, MO) and 0.03% H_2_O_2_. Slides were counterstained with haematoxylin, mounted in Kaiser glycerol and evaluated using light microscopy (Leica, Solms, Germany).

### FACS analysis

After erythrocyte lysis of whole blood, white blood cells were resuspended in PBS containing 1% Fetal Calf Serum (FCS, Sigma, St Louis, MO) and 2.5 mM EDTA. Cells were stained with CD3-AmCyan, CD19-phycoerythrin (PE) (all from BD Bioscience, Sharon, MA), CD14-PerCP-CY5.5 (Biolegend, San Diego, CA), washed, and fluorescence intensities were measured by FACS analysis (BD FacsCanto-II). Viable cells were gated based on FSC/SSC plots (P1), CD3^+^, CD19^+^ and CD14^+^ cells were represented as a percentage of P1.

### Genomic DNA isolation and SNP analysis

For *ATG16L1* SNP status, genomic DNA was isolated from erythrocyte-lysed whole blood using the Wizard Genomic DNA purification Kit from Promega (Madison, WI) as per manufacturers’ instructions and rs2241880 status was determined by KBioscience UK ltd, Hertforshire, UK, using fluorescence based competitive allele-specific PCR.

### qRT-PCR from total RNA

RNA was extracted from whole blood and 300ng was reverse-transcribed with the QuantiTect Reverse Transcription Kit (Qiagen) following manufacturers recommendations. Resulting cDNA was diluted 3-fold, and used in triplicate qRT-PCR for amplification of two housekeeping genes (*RPLP1* forward primer: AGC CTC ATC TGC AAT GTA GGG, reverse primer: TCA GAC TCC TCG GAT TCT TCT TT and *PPIA* forward primer: ATG GTC AAA CCC ACC GTG T, reverse primer TCT GCT GTC TTT GGG ACC TTG TC) and *INPP5D* (forward primer: AAG TGT CGT CTC TCC ACC C, reverse primer: CGG GGA TTC TCG TTT GAA AAA GG), using 2xSYBR green Master Mix (Roche Applied Sciences) on a Roche LightCycler480. Melting curve analysis was performed to confirm the identity of amplified products and assays were performed in duplicate. The 2^nd^ derivative max for each amplification was used to calculate relative expression of SHIP1 according to the ΔΔCt method between healthy control and IBD patients. Notably, ΔCt computations were made using *RPLP* rather than *PPIA* based on its greater abundance (lower Ct values) and reduced variability (lower standard deviations).

### Statistical analysis

Differences in protein expression and activity levels between healthy controls and CD patients were tested by non-parametric test for unpaired samples (Mann Whitney test) using GraphPad Prism 5 software (La Jolla, CA). Three group comparisons of median variation were performed by Kruskal-Wallis non-parametric testing.

#### Ethical considerations

This study was approved by the ethical board of the Erasmus MC, Rotterdam, The Netherlands (protocol MEC-2004-168). Patients in clinical remission and healthy controls were included after written informed consent was obtained.

## Results

### Abrogated SHIP1 activity and protein expression in a subset of CD patients

We set out to investigate SHIP1 expression and activity in isolated peripheral blood mononuclear cells (PBMCs) from a cohort of adult CD patients (mean age 37yr [18–61]), 53% female, see **Table 1**) and healthy controls (mean age 32 yr [24–56], 25% female). As PBMC isolates are a heterogeneous population consisting mainly of lymphocytes (T-cells and B-cells) and monocytes, altered composition of these cell subsets or differences in SHIP1 expression in these subsets could potentially affect total PBMC SHIP1 expression and activity. We first showed that there are minor differences in SHIP1 expression between different cell subsets (**S1 Fig, panel A)**. However, as there were no significant differences between CD patients (n = 15) and healthy controls (n = 10) in the percentage of CD19^+^ B-cells, CD3^+^ T-cells or CD14^+^ monocytes, this is unlikely to affect results (See **S1 Fig, panel B**).

**Fig 1:**
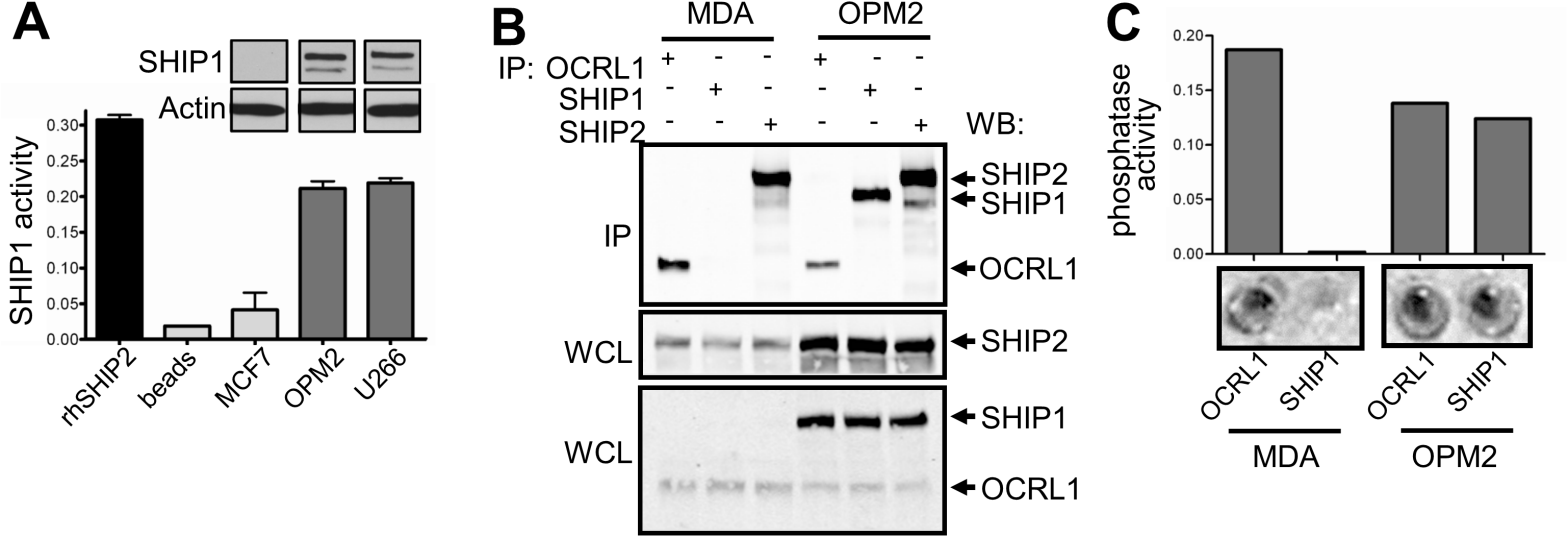
Specific SHIP1 activity measurement. (A) Specificity of the assay was determined by immunoprecipitation (IP) of SHIP1 from multiple myeloma cell lines (U266 and OPM2) that express SHIP1 (see Western blot insert of whole cell lysates) and breast cancer cell line (MCF7) that does not. Phosphatase activity of immunoprecipitates was determined by adding the SHIP1 substrate PtdIns(3,4,5)P_3_, followed by Malachite Green reaction. Human recombinant SHIP2 was used as positive control for the phosphatase assay. Only immunoprecipitates from SHIP1-competent cells show phosphatase activity. (B) OCRL1, SHIP2 or SHIP1 were immunoprecipitated from SHIP1-competent U266 cells and SHIP1-negative MDA-MB-231 (MDA) breast cancer cells. IPs were run on Western blot and probed for SHIP1, SHIP2 and OCRL1 (upper panel). Whole cell lysates (WCL) were also subjected to Western blot analysis of these phosphatases (lower panels). OCRL1 and SHIP2 were precipitated from both MDA-MB-231 and OPM2 cells, whereas SHIP1 was only observed in OPM2, emphasizing specificity of the IP. (C) Malachite Green phosphatase assay (wells shown in lower panels) shows no activity in SHIP1 precipitates from MDA-MB-231 cells, whereas SHIP1 activity was observed in U266 cells. In contrast, OCRL1 phosphatase activity was detected in both cell lines.

**Fig 2:**
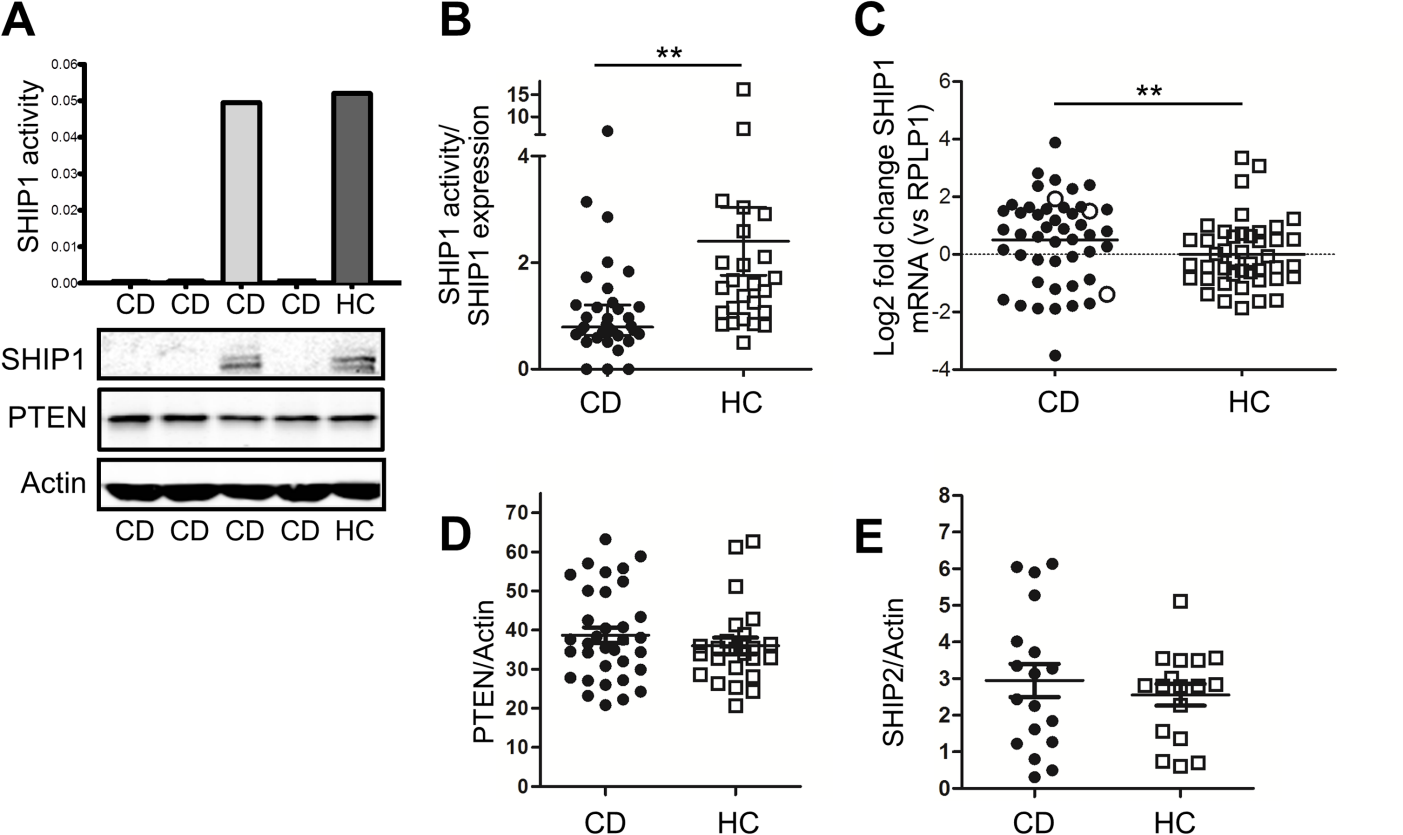
Aberrant SHIP1 activity and expression in adult CD patients. (A) PBMCs from CD patients and healthy controls (HC) were lysed, phosphatase assay performed. Examples of 3 of 5 CD patients with abrogated SHIP1 expression are shown. Lower panels show SHIP1, PTEN and actin protein in the same PBMC lysates of CD patients and healthy controls. Phosphatase activity in these patients was tested in duplicate, with identical results. (B) Correction of SHIP1 phosphatase activity for the amount of SHIP1 protein in the lysates shows that intrinsic SHIP1 enzymatic activity is significantly lower in CD patients compared to HC. (C) Increased mRNA and protein (examples in S2A Fig) expression compensates for the reduced intrinsic enzymatic activity (CD patients [n = 47] vs healthy controls [n = 42], 1.68 fold increase, p = 0.0042). RNA was available of three of the five SHIP1-deficient patients (open circles). (C) PTEN protein expression in PBMC lysates from CD patients and healthy controls (HC) were determined by Western blot analysis, and corrected for Actin levels in the same samples. Quantification of individual experiments, including mean ±SD are shown. (D,E) PTEN (D) and SHIP2 (E) protein expression in PBMC lysates from CD patients and healthy controls (HC) were determined by Western blot analysis, and corrected for Actin levels in the same samples. Quantification of individual experiments, including mean ±SD are shown (examples in Fig 2A lower panel and S2B Fig).

SHIP1 enzymatic activity was determined by precipitating endogenous SHIP1 from cells and incubating precipitates with the SHIP1 substrate PtdIns(3,4,5)P_3_. The specificity and selectivity of this approach was shown by comparing phosphatase activity in SHIP1-deficient and proficient cell lines (**Fig 1A–1C),** and the quantitative range of this assay in peripheral blood mononuclear cells (PBMCs) was determined (**S1 Fig, panel C**). SHIP1 activity was subsequently determined in PBMCs from CD patients (n = 34) and healthy controls (n = 25). No significant difference in total cellular SHIP1 activity was observed between patients and controls (0.15±0.01 vs 0.18±0.02 AU p = 0.175). However, the SHIP1 activity measured in cells is of course dependent on the amount of SHIP1 protein present in the cell lysates. Interestingly, SHIP1 protein levels were at or below detection level in 5 patients (14.7%) (examples shown in **Fig 2A lower panel**). No obvious clinical differences were noted between these and other CD patients (#5, 6, 8, 13 and 28, see **S1 Table**). As expected, total cellular SHIP1 activity was abrogated in patients in whom SHIP1 protein expression was lost (**Fig 2A upper panel**). In the remainder of patients, SHIP1 protein was detected and even increased (5.8±0.6 vs 3.8±0.5, p = 0.0084, see examples in **S2 Fig, panel A**), implicating that intrinsic enzymatic activity of the SHIP1 protein is reduced as compared to healthy controls (SHIP1 activity/SHIP1 expression 1.14±0.2 for CD vs 2.40±0.6 for HC, p = 0.0006, **Fig 2B**). In line with the increased protein expression, we also observed an enhanced mRNA expression in PBMCs from CD patients compared to healthy controls (**Fig 2C**). MRNA was available of 3 of the 5 patients with abrogated SHIP1 expression, and was not absent in these patients, suggesting that a post translational mechanism accounts for the impaired SHIP1 expression in these patients. No differences in protein expression levels of the inositol phosphatases SHIP2 and PTEN were observed (see **Fig 2D and 2E, examples in Fig 2A and S2 Fig, panel B**).

### SHIP1 activity and expression are not related to ATG16L1 polymorphism

**Fig 3:**
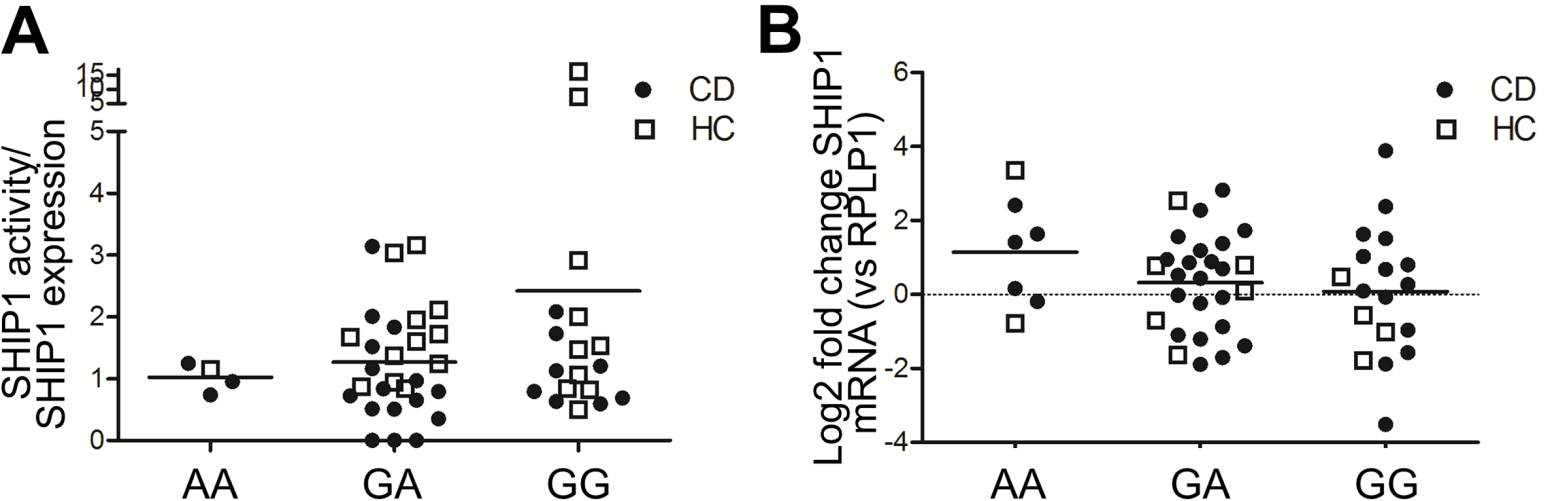
SHIP1 expression and activity are independent of *ATG16L1* SNP status. ATG16L1 rs2241880 SNP status was determined for those subjects for whom genomic DNA was available. Groups were stratified according to protective (AA), heterozygous (AG) or risk (GG) alleles. (A) SHIP1 activity corrected for total amount of SHIP1 present in the lysates is shown. The number of risk alleles does not affect SHIP1 intrinsic activity levels. (B) SHIP1 mRNA levels corrected for RPLP1 mRNA levels are shown for patients and controls, stratified according to *ATG16L1* SNP status. The number of *ATG16L1* risk alleles does not affect SHIP1 mRNA expression.

The *INPP5D* gene encoding SHIP1 lies adjacent to the CD-risk gene *ATG16L1*. Large genome wide association studies have shown the SNPs rs2241880 in *ATG16L1* to be strongly associated with CD development[14]. Ngoh *et al* found a correlation between *ATG16L1* SNP status and SHIP1 activity and expression in their cohort[9]. We therefor stratified our adult CD patients and healthy controls for whom genomic DNA was available (total n = 48) by the number of *ATG16L1* risk alleles. The number of risk alleles carried by the donor did not affect either SHIP1 activity (**Fig 3A**) or mRNA expression (**Fig 3B**).

## Discussion

Genetic ablation of SHIP1 causes a Crohn’s-like phenotype in mice [4,5], and it was recently demonstrated that SHIP1 protein expression and activity is decreased in PMBCs and biopsies from CD patients, which was linked to the presence of the *ATG16L1* risk allele rs2441880 [9,10]. It has been suggested that rs12994997, a SNP in close linkage disequilibrium with rs2441880, could be an expression quantitative trait locus for *INPP5D* [15]. In the current manuscript, we demonstrate that while in the majority of adult CD patients intrinsic SHIP1 activity is decreased, this is unaffected by the number of *ATG16L1* risk alleles. In addition, an increase rather than a decrease in SHIP1 mRNA and protein expression was observed, which corrects total cellular levels of SHIP1 activity. Interestingly, Arijs *et al*. recently also reported increased mRNA expression of SHIP1 in mucosal biopsies from CD patients, although phosphatase activity was not reported[8]. Differences between our study and that of Ngoh *et al*. may be explained by the different cohorts studied (age, treatment, disease location) or different SHIP phosphatase assays used.

It is becoming apparent that several individual triggers need to combine for IBD to become manifest, and that not all patients suffer from the same molecular defects contributing to their disease. Thus, SHIP1 deficiency is also not expected to contribute to disease in all patients. It is therefore of interest that our study demonstrates that SHIP1 protein expression is abrogated in only a subset of CD patients, resulting in greatly decreased total cellular SHIP1 activity and potentially contributing to disease pathology in this subset of CD patients. Expression of SHIP1 mRNA was not abrogated in these patients, implying that a translational or post-translational mechanism contributes to the decrease in SHIP1 protein levels in this subset of patients.

The molecular mechanisms contributing to the dichotomy in SHIP1 expression remain unclear. Large genome wide association studies have now identified over 140 genetic loci associated with the development of CD. However, as yet only 10% of total disease variance has been explained [15], and rare variants not previously identified by GWAS are now being recognized [16]. The *ATG16L1* variant rs2241880 lies in a region (IBD10, 2q37 locus) that also contains other SNPs that co-segregate with disease, and it is possible that genes other than *ATG16L1* at this locus may contribute to pathogenesis of CD. This notion is not unprecedented, as a similar phenomenon is observed at chromosome 1 (IBD risk locus 7), where several genes within this 1p36 locus confer risk of CD (genes encoding Runx3, Casp9, p110δ). In addition, modulation of SHIP1 mRNA by miR155 or protein by ubiquitibation and proteasomal degradation could provide two other potential mechanisms for posttranslational modification of the SHIP1 protein [12,17].

*In toto*, our study shows that SHIP1 activity in PBMCs is decreased in adult CD patients either through reduced intrinsic enxymatic activity or reduced protein expression, and we propose that in addition to *ATG16L1*, SHIP1 may contribute to the risk conferred by the 2q37 CD risk locus.

## Supporting information

S1 Table Clinical details of individual patients for whom SHIP1 activity was measured. (DOC)

S1 Fig SHIP1 activity measurement is affected by amount of SHIP1 protein present in the lysate, but does not depend on PBMC cell distribution. (A) The main components of PBMCs constitute T-cells, B-cells and monocytes and these subsets were isolated from PBMCs by positive selection with CD3-, CD19- and CD14-microbeads respectively, followed by manual MACS sorting. Isolated fractions were lysed, proteins separated by Western blot analysis and immunoblotted for SHIP1 protein. Equal loading was confirmed by reprobing blots with antibodies against Actin. Lower panel: cytospots were made from whole PBMC fraction and stained with SHIP1 antibodies, showing no differences between different cell types. Specificity of the antibody was confirmed by negative staining of cytospots of Caco2 cells. (B) Differences in PBMC subsets could potentially affect the amount of SHIP1 protein measured. However. no differences in the percentages of CD19^+^ B-cells, CD3^+^ T-cells or CD14^+^ monocytes were observed between CD patients (n = 15 and healthy controls (HC, n = 10) by FACS analysis of PBMCs (mean ±SEM). (C) OPM2 cells were lysed, and dilutions with increasing amounts of total protein were subjected to SHIP1 phosphatase assay. Dilutions were also subjected to Western blot analysis, and total SHIP1 levels quantified by Odyssey 3 software. SHIP1 activity and SHIP1 expression patterns show excellent correlation, demonstrating that the amount of SHIP1 protein input determines SHIP1 activity measured.
(TIF)

S2 Fig Examples of SHIP1 activity and SHIP1, SHIP2 and PTEN expression in CD patients and healthy controls. (A) Representative Western blot examples of patients (CD) and healthy controls (HC) showing increased SHIP1 expression, which did not correspond with SHIP1 activity levels. (B) Representative Western blot examples showing no difference in PTEN or SHIP2 expression between CD patients and healthy controls (HC).
(TIF)

## References

[1] Geier SJ, Algate PA, Carlberg K, Flowers D, Friedman C, Trask B, et al The human SHIP gene is differentially expressed in cell lineages of the bone marrow and blood. Blood. 1997;89: 1876–1885. 9058707

[2] Kerr WG. Inhibitor and activator: dual functions for SHIP in immunity and cancer. AnnNYAcadSci. 2011;1217: 1–17. Available: http://www.ncbi.nlm.nih.gov/pubmed/21155837 10.1111/j.1749-6632.2010.05869.x PMC4515353 21155837

[3] Rauh MJ, Ho V, Pereira C, Sham A, Sly LM, Lam V, et al SHIP represses the generation of alternatively activated macrophages. Immunity. 2005;23: 361–374. Available: http://www.ncbi.nlm.nih.gov/pubmed/16226502 doi: 10.1016/j.immuni.2005.09.003 16226502 10.1016/j.immuni.2005.09.003

[4] Kerr WG, Park MY, Maubert M, Engelman RW. SHIP deficiency causes Crohn’s disease-like ileitis. Gut. 2011;60: 177–188. Available: http://www.ncbi.nlm.nih.gov/pubmed/20940287 doi: 10.1136/gut.2009.202283 20940287 10.1136/gut.2009.202283 PMC3022365

[5] McLarren KW, Cole AE, Weisser SB, Voglmaier NS, Conlin VS, Jacobson K, et al SHIP-deficient mice develop spontaneous intestinal inflammation and arginase-dependent fibrosis. AmJPathol. 2011;179: 180–188. Available: http://www.ncbi.nlm.nih.gov/pubmed/21640975 10.1016/j.ajpath.2011.03.018 PMC3123870 21640975

[6] Ghansah T, Paraiso KH, Highfill S, Desponts C, May S, McIntosh JK, et al Expansion of myeloid suppressor cells in SHIP-deficient mice represses allogeneic T cell responses. JImmunol. 2004;173: 7324–7330. Available: http://www.ncbi.nlm.nih.gov/pubmed/15585856 15585856 10.4049/jimmunol.173.12.7324

[7] Maxwell MJ, Srivastava N, Park M-Y, Tsantikos E, Engelman RW, Kerr WG, et al SHIP-1 deficiency in the myeloid compartment is insufficient to induce myeloid expansion or chronic inflammation. Genes Immun. 2014;15: 233–40. doi: 10.1038/gene.2014.9 24598798 10.1038/gene.2014.9

[8] Arijs I, De Hertogh G, Lemmens B, Van der Goten J, Vermeire S, Schuit F, et al Intestinal expression of SHIP in inflammatory bowel diseases. Gut. 2011/11/05. 2012;61: 956–957. doi: 10.1136/gutjnl-2011-301256 22052065 10.1136/gutjnl-2011-301256

[9] Ngoh EN, Brugger HK, Monajemi M, Menzies SC, Hirschfeld AF, Del Bel KL, et al The Crohn’s disease-associated polymorphism in ATG16L1 (rs2241880) reduces SHIP gene expression and activity in human subjects. Genes Immun. 2015;16: 452–61. doi: 10.1038/gene.2015.30 26226011 10.1038/gene.2015.30

[10] Ngoh EN, Weisser SB, Lo Y, Kozicky LK, Jen R, Brugger HK, et al Activity of SHIP, Which Prevents Expression of Interleukin-1β, is Reduced in Patients with Crohn’s Disease. Gastroenterology. 2015; doi: 10.1053/j.gastro.2015.09.049 26481854 10.1053/j.gastro.2015.09.049

[11] Henderson P, van Limbergen JE, Wilson DC, Satsangi J, Russell RK. Genetics of childhood-onset inflammatory bowel disease. Inflamm Bowel Dis. 2011;17: 346–361. doi: 10.1002/ibd.21283 20839313 10.1002/ibd.21283

[12] Brooks R, Fuhler GM, Iyer S, Smith MJ, Park MY, Paraiso KH, et al SHIP1 inhibition increases immunoregulatory capacity and triggers apoptosis of hematopoietic cancer cells. JImmunol. 2010;184: 3582–3589. Available: http://www.ncbi.nlm.nih.gov/pubmed/20200281 20200281 10.4049/jimmunol.0902844 PMC4123216

[13] Fuhler GM, Tyl MR, Olthof SG, Lyndsay DA, Blom N, Vellenga E. Distinct roles of the mTOR components Rictor and Raptor in MO7e megakaryocytic cells1. EurJHaematol. 2009; Available: http://www.ncbi.nlm.nih.gov/pubmed/19341427 10.1111/j.1600-0609.2009.01263.x 19341427

[14] Rioux JD, Xavier RJ, Taylor KD, Silverberg MS, Goyette P, Huett A, et al Genome-wide association study identifies new susceptibility loci for Crohn disease and implicates autophagy in disease pathogenesis. NatGenet. 2007;39: 596–604. Available: http://www.ncbi.nlm.nih.gov/pubmed/17435756 10.1038/ng2032 PMC2757939 17435756

[15] Jostins L, Ripke S, Weersma RK, Duerr RH, McGovern DP, Hui KY, et al Host-microbe interactions have shaped the genetic architecture of inflammatory bowel disease. Nature. 2012/11/07. 2012;491: 119–124. doi: 10.1038/nature11582 23128233 10.1038/nature11582 PMC3491803

[16] Muise AM, Xu W, Guo CH, Walters TD, Wolters VM, Fattouh R, et al NADPH oxidase complex and IBD candidate gene studies: identification of a rare variant in NCF2 that results in reduced binding to RAC2. Gut. 2011/09/09. 2012;61: 1028–1035. doi: 10.1136/gutjnl-2011-300078 21900546 10.1136/gutjnl-2011-300078 PMC3806486

[17] O’Connell RM, Chaudhuri AA, Rao DS, Baltimore D. Inositol phosphatase SHIP1 is a primary target of miR-155. Proc Natl Acad Sci U S A. 2009/04/11. 2009;106: 7113–7118. doi: 10.1073/pnas.0902636106 19359473 10.1073/pnas.0902636106 PMC2678424

