# Supplementary material for: Analysis of SHIP1 expression and activity in Crohn’s disease patients
Source: PLoS One. 2017 Aug 2;12(8):e0182308. doi: 10.1371/journal.pone.0182308 (PMC5540589; doi:10.1371/journal.pone.0182308)
Supplement: S1 Table — (DOC) [file pone.0182308.s001.doc]

|  | Gender | Age | Age at diagnosis | Duration of disease | Disease location | ATG16L1 rs2241880 | Resection | CRP mg/L | Disease behaviour | Treatment | Other |
| --- | --- | --- | --- | --- | --- | --- | --- | --- | --- | --- | --- |
| 1 | F | 56 | 24 | 32 | Ileocolonic | GA | No | 2 | B3 | Adalimumab |  |
| 2 | F | 41 | 26 | 15 | Ileocolonic | Nd | No | <1 | B1 | Adalimumab, Azathioprine |  |
| 3 | M | 18 | 17 | 1 | Ileal | GA | No | 1 | B1 | Adalimumab |  |
| 4 | F | 50 | 45 | 5 | Colonic | Nd | No | <1 | B1 | Adalimumab |  |
| 5 | F | 51 | 39 | 12 | Colonic | GA | No | <1 | B3 | Azathioprine, 5-ASA |  |
| 6 | F | 34 | 23 | 11 | Ileal | GA | Yes | 1 | B2 |  |  |
| 7 | F | 43 | 33 | 10 | Ileal | GG | Yes | <1 | B2 |  |  |
| 8 | F | 54 | 44 | 10 | Ileocolonic | GA | No | 2 | B2 | Azathioprine, 5-ASA |  |
| 9 | F | 42 | 21 | 21 | Ileocolonic | GG | Yes | 3 | B3 | Adalimumab, Budesonide |  |
| 10 | M | 40 | 19 | 21 | Ileocolonic L3+ L4 | GG | Yes | <1 | B1 | Adalimumab | Thrombosis |
| 11 | M | 24 | 16 | 8 | Ileocolonic | Nd | Yes | 6 | B2 | Infliximab |  |
| 12 | F | 33 | 29 | 4 | Colonic | GA | No | 2 | B1 | Adalimumab | Sacro-ileitis |
| 13 | M | 19 | 15 | 5 | Ileal | Nd | Yes | 2 | B2 | Azathioprine |  |
| 14 | F | 31 | 20 | 11 | Ileocolonic | GG | Yes | <1 | B2 | Azathioprine |  |
| 15 | M | 33 | 14 | 19 | Ileocolonic | GG | Yes | 2 | B1 |  |  |
| 16 | M | 36 | 20 | 14 | Ileal | GA | Yes | 2 | B2 | Prednison, MTX |  |
| 17 | M | 42 | 36 | 6 | Colonic | Nd | No | 1 | B1 | 5-ASA | H.pylori |
| 18 | M | 47 | 31 | 16 | Colonic | GA | Yes | 3 | B3 | Adalimumab | Sacro-ileitis |
| 19 | F | 50 | 28 | 22 | Colonic | GG | No | Nd | B1 | Azathioprine |  |
| 20 | M | 61 | 55 | 6 | Ileocolonic | Nd | Yes | <1 | B2 | Adalimumab |  |
| 21 | M | 21 | 10 | 11 | Ileocolonic | GA | No | 2 | B2 | Adalimumab, 5-ASA |  |
| 22 | F | 26 | 23 | 3 | Ileal | Nd | No | Nd | B1 |  |  |
| 23 | F | 39 | 19 | 20 | Colonic | Nd | No | Nd | B1 |  | Sclerosing Cholangitis |
| 24 | M | 30 | 20 | 10 | Ileal | AA | Yes | Nd | B3 |  |  |
| 25 | M | 48 | 21 | 27 | Ileocolonic | GA | Yes | 9 | B1 | Prednison, 6-MP | Colorectal cancer |
| 26 | F | 29 | 18 | 11 | Ileocolonic +L4 | GG | No | 2 | B3 | Adalimumab |  |
| 27 | F | 32 | 29 | 3 | Ileal | GG | No | Nd | B1 | Azathioprine |  |
| 28 | F | 23 | 15 | 8 | Colonic | GA | No | 65 | B1 | Infliximab, azathiop, 5-ASA |  |
| 29 | M | 35 | 21 | 14 | Colonic | GA | No | Nd | B1 |  |  |
| 30 | M | 31 | 25 | 6 | Colonic | AA | No | 18 | B3 | Adalimumab, MTX |  |
| 31 | F | 22 | 18 | 4 | Ileocolonic | AA | Yes | 6 | B1 |  |  |
| 32 | M | 47 | 28 | 19 | Ileocolonic | GA | Yes | <1 | B2 | Adalimumab |  |
| 33 | F | 37 | 29 | 8 | Ileocolonic | Nd | Yes | 22 | B2 | Adalimumab, budesonide |  |
| 34 | M | 45 | 18 | 27 | Ileal +L4 | GA | No | 2 | B3 |  |  |

**Supplementary Table 1. Clinical details of individual patients for which SHIP1 activity was measured.** 5-ASA: 5-aminosalicylic acid; MTX: methotrexate
