# Supplementary figures and images for: Analysis of SHIP1 expression and activity in Crohn’s disease patients
Source: PLoS One. 2017 Aug 2;12(8):e0182308. doi: 10.1371/journal.pone.0182308 (PMC5540589; doi:10.1371/journal.pone.0182308)

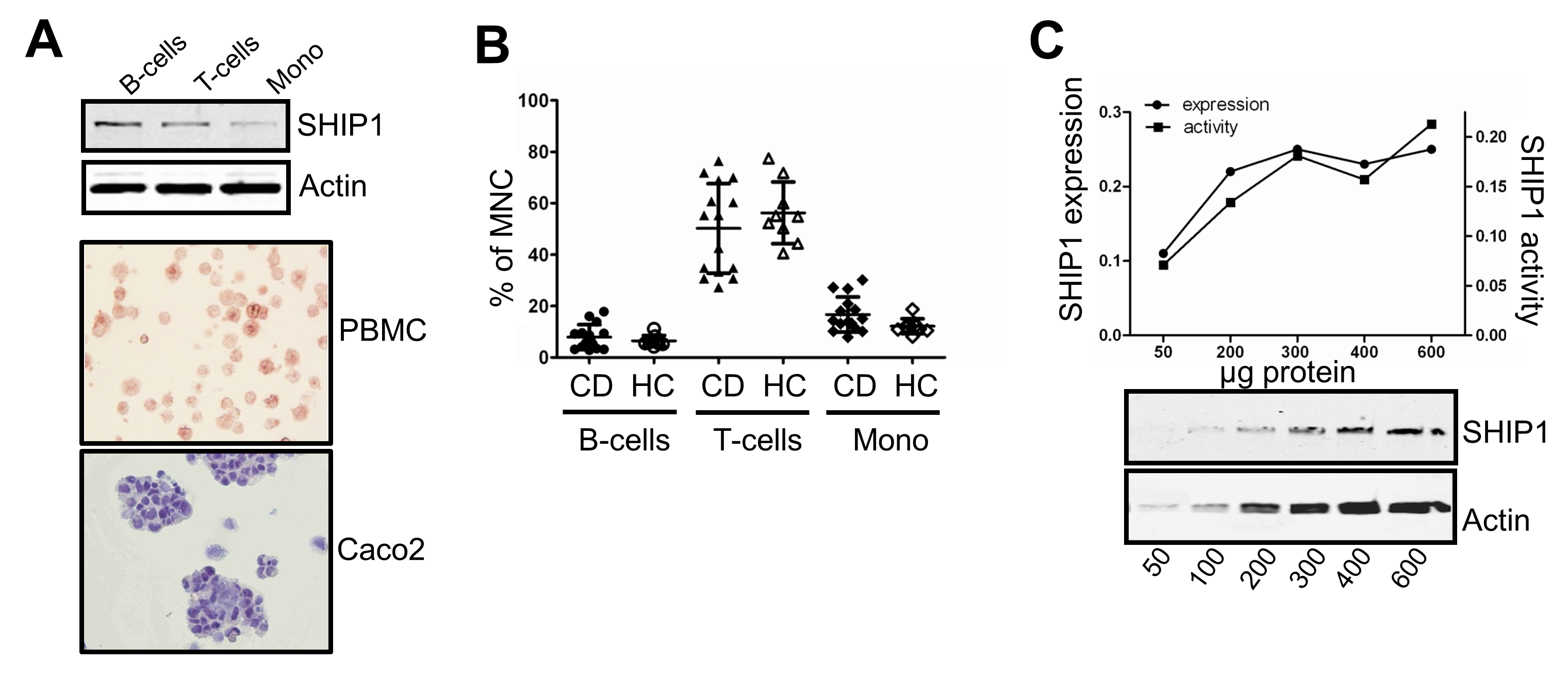

Supplement: S1 Fig — (A) The main components of PBMCs constitute T-cells, B-cells and monocytes and these subsets were isolated from PBMCs by positive selection with CD3-, CD19- and CD14-microbeads respectively, followed by manual MACS sorting. Isolated fractions were lysed, proteins separated by Western blot analysis and immunoblotted for SHIP1 protein. Equal loading was confirmed by reprobing blots with antibodies against Actin. Lower panel: cytospots were made from whole PBMC fraction and stained with SHIP1 antibodies, showing no differences between different cell types. Specificity of the antibody was confirmed by negative staining of cytospots of Caco2 cells. (B) Differences in PBMC subsets could potentially affect the amount of SHIP1 protein measured. However. no differences in the percentages of CD19+ B-cells, CD3+ T-cells or CD14+ monocytes were observed between CD patients (n = 15 and healthy controls (HC, n = 10) by FACS analysis of PBMCs (mean ±SEM). (C) OPM2 cells were lysed, and dilutions with increasing amounts of total protein were subjected to SHIP1 phosphatase assay. Dilutions were also subjected to Western blot analysis, and total SHIP1 levels quantified by Odyssey 3 software. SHIP1 activity and SHIP1 expression patterns show excellent correlation, demonstrating that the amount of SHIP1 protein input determines SHIP1 activity measured. (TIF) [file pone.0182308.s002.tif]

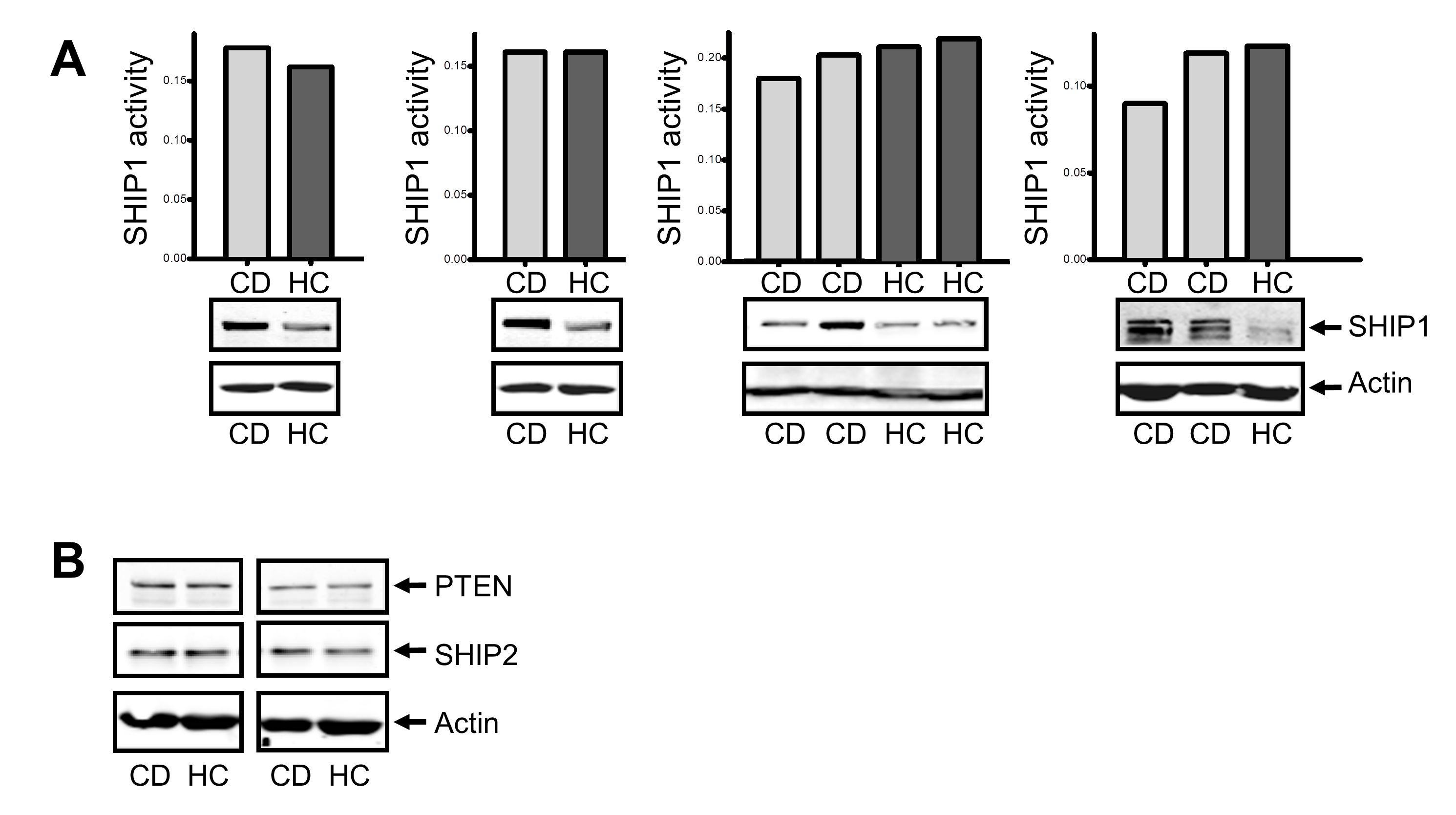

Supplement: S2 Fig — (A) Representative Western blot examples of patients (CD) and healthy controls (HC) showing increased SHIP1 expression, which did not correspond with SHIP1 activity levels. (B) Representative Western blot examples showing no difference in PTEN or SHIP2 expression between CD patients and healthy controls (HC). (TIF) [file pone.0182308.s003.tif]
